# Different Temperature Storage Conditions and Packaging Types Affects Colour Parameters, Amino Acid Composition, Microbial Contamination, and Key Bioactive Molecules of *Moringa oleifera* Lam. Powder

**DOI:** 10.3390/molecules30204048

**Published:** 2025-10-11

**Authors:** Ashwell R. Ndhlala, Gladness T. Ngobeni, Rofhiwa Mulaudzi, Sogolo L. Lebelo

**Affiliations:** 1 Department of Life and Consumer Sciences, College of Agriculture and Environmental Sciences, University of South Africa, Private Bag X6, Florida 1710, South Africa; 66646758@mylife.unisa.ac.za (G.T.N.); rofhim@unisa.ac.za (R.M.); 2 Green Biotechnologies Research Centre of Excellence, School of Agriculture and Environmental Sciences, University of Limpopo, Private Bag X1106, Sovenga 0727, South Africa

**Keywords:** food preservation, microbial contamination, nutritional quality, phytochemical composition, shelf life

## Abstract

*Moringa oleifera*, renowned for its medicinal potency, was investigated to discern the impact of varying storage temperatures (4 °C, 25 °C, 40 °C) and light conditions (dark and light) on the quality attributes of its leaf powder during a 12-month storage period. The study encompassed comprehensive analyses of phytochemical levels, nutritional properties, microbial contamination, and colour changes in response to these diverse storage environments. The lightness L* colour value changed significantly (40 to 60) from baseline tests when stored at 40 °C in transparent packaging. Results highlighted distinct variations in phytochemical composition and nutritional content based on the interplay between temperature and light conditions. Lower temperatures, particularly 4 °C, in both dark and light environments, demonstrated superior preservation of bioactive compounds, with mean values for quercetin-3-rutinoside of 3.34 µg/g and 3.19 µg/g, respectively; both are significantly higher compared to other treatments (*p* < 0.05). This trend was also observed for rutin, chlorogenic acid, and quercetin. Conversely, higher temperatures (25 °C, 40 °C) coupled with light exposure hastened degradation, notably impacting phytochemical stability. Microbial proliferation was evident in elevated temperatures, indicating potential safety risks. Further observations unveiled significant colour changes within the leaf powder, notably influenced by storage temperatures and light exposure. Lower temperatures exhibited diminished colour alterations compared to higher temperatures, underscoring their impact on product quality. This study underscores the critical role of controlled storage conditions, especially cooler temperatures and reduced light exposure, in maintaining the potency and quality of *M. oleifera* leaf powder. Recommendations advocate for stringent temperature control (preferably 4 °C) and light shielding during storage to uphold phytochemical stability and mitigate microbial proliferation. While this study provides valuable insights into temperature-mediated alterations, future research avenues should delve deeper into elucidating the underlying mechanisms of colour changes and long-term temperature effects on phytochemical and nutritional integrity.

## 1. Introduction

Medicinal plants form the primary base of health care, not only for rural communities but also for urban communities, and they also contribute as a source of income [1]. Over the past twenty years, there has been a drastic increase in interest in traditional systems of medicines and herbal medicines not just in developed countries but also in developing countries. Rapid commercialization of medicinal herbs has seen a significant growth in the economy [2,3]. Medicinal plants are therapeutics that are easily accessible and affordable. As such, over 80% of the world’s population rely on the use of medicinal plants as natural remedies [4].

*Moringa oleifera* Lam. is known as a multipurpose plant with a vast variety of potential nutritional use [5,6]. *Moringa oleifera* is abundant in essential vitamins and minerals, with the leaves being the most nutritious part of the plant. *Moringa oleifera* leaves have shown to be an excellent source of vitamin A, magnesium, calcium, vitamin B6, vitamin C, and beta carotene pro-vitamin A [7,8]. Previous studies have demonstrated that *M. oleifera* leaves have greater amounts of Vitamin A than in carrots, more calcium than in milk, as well as more iron than in spinach [9]. *Moringa oleifera* is also known to be an excellent source of vitamin C, which is contained more in the leaves than in oranges, as well as more potassium than in a banana. The quality of protein in the *M. oleifera* leaves is similar to that found in milk and eggs [9].

*Moringa oleifera* leaf contains amino acids, which are essential building blocks of proteins and phytochemical compounds that have important biological activities [10]. The leaves can be consumed in many ways: either fresh, cooked, dried leaf, or crushed into a powder [11]. Dried *M. oleifera* leaves are expected to retain their nutrient content. *Moringa oleifera* leaves can be dried and stored for a period beyond six months depending on the conditions. It is advisable to store the food in sealed packaging materials when dried with a required moisture content. To prevent microbiological contamination or moisture in dried products, packaging should be an integral part of food processing and preservation methods, as it depends on the appropriate packaging [12,13].

In various regions of South Africa, *M. oleifera* cultivation serves as a vital source of income and food security within communities due to its nutritional significance. As the demand for *M. oleifera* continues to rise for commercial purposes, ensuring proper storage becomes imperative to uphold its quality and prevent phytochemical degradation. Phytochemicals act as natural compounds devoid of nutritional value and function independently or in conjunction with vitamins and other nutrients to combat diseases [14]. Many phytochemicals exhibit potent biological activities. Extended storage durations may lead to the depletion of both phytochemicals and some nutrients in plant material, potentially compromising their quality and diminishing their value. The challenge of storage is compounded by diverse environmental settings and economic constraints surrounding planting, harvesting, storage, and packaging.

Critical factors influencing storage include packaging methods and storage temperatures, pivotal in enhancing shelf life and preserving quality [15]. Improper drying or storage of *M. oleifera* powder can foster mould or mildew growth, posing issues ranging from unpleasant odours to potential harm [12]. Implementing sound processing practices is essential to minimize microbial contamination, significantly impacting the overall quality and safety of medicinal products. Understanding the optimal post-harvest procedures, including drying, appropriate packaging, and storage methods, is crucial to maintain the quality of these plants and prevent degradation. Improper handling, inadequate drying, and subpar storage conditions can profoundly impact the biological, chemical, and physical composition of the plants, leading to unfavourable microbial alterations [16,17]. The aim of this study was to evaluate how varying storage temperatures and light exposure would affect the phytochemical levels, nutritional properties, and microbial contamination in *M. oleifera* leaf powder. This will inform the optimum product storage conditions as well as the correct processed material for product development.

## 2. Results and Discussion

### 2.1. Colour

#### 2.1.1. Lightness (L*)

**Figure 1:**
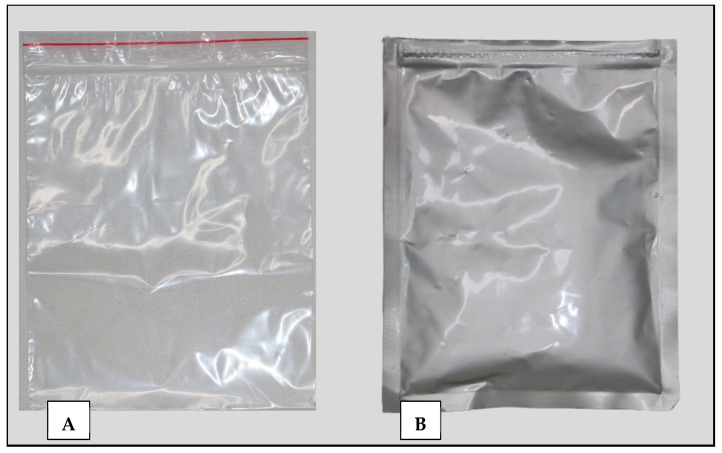
Packaging types used to store *Moringa oleifera* leaf powder. (**A**) transparent packaging; (**B**) opaque packaging.

**Figure 2:**
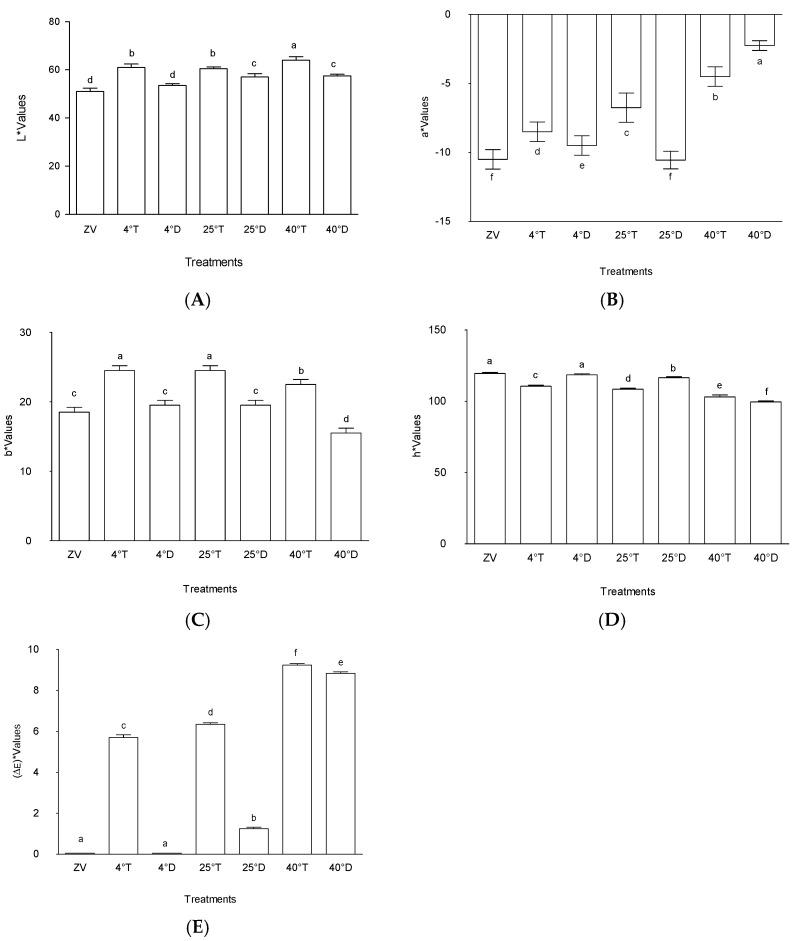
Effect of packaging and temperature treatment on (**A**) lightness L* colour, (**B**) a* colour, (**C**) b* colour, (**D**) h* colour, and (**E**) colour differences (ΔE) values in *Moringa oleifera* leaf powder. ZV—zero-value baseline results of material analyzed before storage; 4 °T—storage at 4 °C with transparent packaging; 4 °D—storage at 4 °C opaque packaging; 25 °T—storage at 25 °C with transparent packaging; 25 °D—storage at 25 °C with opaque packaging; 40 °T—storage at 40 °C with transparent packaging; 40 °D—storage at 40 °C with opaque packaging. *n* = 6 per treatment. Results are expressed as the mean values ± standard error (*n* = 6) including control; bars in the same column marked with different letters indicate significance difference at *p* ≤ 0.05.

Figure 1 depicts the types of storage packaging used in the study, vis a vis, transparent plastic and opaque silver zipper bags. The effects of different packaging type and light exposure on the colour of *M. oleifera* leaf powder are shown in Figure 2. The L* value represents the degree of lightness to darkness, a* represents the degree of redness and greenness, and the b* value represents the yellowness and blueness. The colour parameters (L*, a*, ΔE) were used to determine the most suitable frequency for the study. These values were crucial in understanding the influence of the treatments to colour changes on the powder samples.

The colour analysis showed storage at 40 °C with transparent packaging having a noticeably higher level of lightness (L*) than the other experimental treatments (*p* < 0.05), which meant a high rate of colour fading (Figure 2). On the other hand, storage at 4 °C in a transparent packaging (Figure 1A) and storage at 25 °C in a transparent packaging showed somewhat moderate changes in L* values, which signified moderate changes in colour over time. Storage of *M. oleifera* at 4 °C in an opaque packaging (Figure 1B) had lower Lightness L* values which were not very different from the initial readings taken at day zero of the experiment, which meant little colour changes.

The outcomes observed among the various treatments denote intriguing patterns in the context of colour attributes within differing packaging and temperature conditions. Treatment A40T, characterized by transparent packaging at 40 °C, notably exhibited significantly higher lightness (L*) in comparison to the other treatments investigated. This observation implies that under the specified conditions, the colour of the product experienced a perceptible increase in brightness. Such an outcome could indicate potential alterations in the visual appearance or perceived quality of the product due to this specific packaging and temperature combination. Moreover, the treatments stored at 40 °C with transparent packaging and stored at 25 °C with transparent packaging also demonstrated relatively higher L* values, though without a discernible statistical variance between them (Figure 2). This suggests a consistent trend in the colour enhancement effect, albeit not markedly distinguishable at the tested significance level. This finding underscores the influence of transparency across varied temperature conditions on the product’s colour characteristics. Previous research has noted the impact of light presence on enhancing product lightness [18]. In terms of food storage for *M. oleifera* powder, an increase in the L* value representing lightness could be due to various factors. Exposure to light, especially in improper storage conditions, might trigger photochemical reactions that alter pigments or colour compounds present in the leaf powder. This exposure can lead to degradation or breakdown of these compounds, resulting in a lighter appearance of the powder and an increase in the L* value.

Conversely, treatments stored at 4 °C with opaque packaging and control (zero value) displayed lower L* values, indicating a comparatively reduced lightness in these conditions. Despite exhibiting a degree of variability, the observed differences in lightness between storage at 4 °C with opaque packaging and control (zero value) were not statistically significant within the tested parameters (*p* < 0.05). This lack of statistical distinction suggests that the dark packaging at 4 °C did not significantly alter the product’s colour attributes compared to its initial state control (zero value). Wang et al. [19] suggested that utilizing dark packaging might shield products from photo-oxidation, potentially aiding in preserving their qualities.

Overall, these findings underscore the substantial impact of packaging transparency and temperature variations on the colour attributes of the product. The significant increase in lightness observed in storage at 40 °C with transparent packaging compared to the other treatments suggests a potential influence of transparent packaging at higher temperatures on the visual perception and potentially the perceived quality of the product. However, further detailed analyses and complementary investigations may be warranted to comprehensively elucidate the underlying mechanisms driving these observed colour alterations and their broader implications for product quality and consumer perception.

#### 2.1.2. Colour Redness (a*)

The observed outcomes regarding the “a*” values across the treatments offer valuable insights into the colour attributes influenced by varying experimental conditions (Figure 2). The significant elevation in “a*” values within treatments stored at 40 °C with opaque packaging and stored at 40 °C with transparent packaging, when juxtaposed with other treatments, aligns with the notion that these specific conditions—presumably associated with opaque packaging at 40 °C and transparent packaging at 40 °C—contribute substantially to heightened colour attributes, particularly in the red–green axis.

In comparison to storage at 25 °C with opaque packaging, control (zero value), and storage at 4 °C with opaque, the treatment stored at 25 °C with transparent packaging has a noticeably higher “a*” value. This indicates that, in comparison to the previously described treatments, transparent packaging at 25 °C highlights the red–green axis in colour characteristics more, highlighting the complex relationship between temperature and packaging transparency and colour development.

These results are consistent with earlier studies investigating the impact of packaging and ambient factors on colour characteristics. Analogous research has emphasized how temperature variations and the transparency of packing materials influence colour qualities. For example, the study by Phahom et al. [20] supported the patterns seen in the treatment stored at 40 °C with opaque packaging and storage at 40 °C with transparent packaging by demonstrating the relationship between rising temperatures and an increase in particular colour characteristics. Results obtained by Krah and Krisnadi [21] also highlighted the way that transparent packing materials can amplify colour features, which is similar to the higher “a*” value that the treatment stored at 25 °C with transparent packaging showed in comparison to other conditions.

Heat and oxygen during storage encourage chlorophyll colour change in dried leaves [22]. Chlorophyll in leaves can experience oxidation, hydrolysis, and isomerization after harvest. Chlorophyll’s magnesium atom is swapped out for two hydrogen atoms, which causes the colour to shift from brilliant green to olive green [20,23]. The observed differences in “a*” values between treatments highlight how temperature and the transparency of the packing material affect particular colour axes, adding to our understanding of how colour develops and stabilizes in a variety of product packaging scenarios.

#### 2.1.3. Colour Yellowness (b*)

The results of this investigation clearly show that different treatment circumstances have a significant impact on “b*” values, which are a crucial parameter that represent the yellow–blue axis within colour characteristics (Figure 2). Treatment storage at 4 °C with transparent packaging and storage at 25 °C with transparent packaging were found to have the highest “b*” values, indicating a significant increase along the yellow–blue axis. In particular, the treatment stored at 4 °C with transparent packaging was distinguished from the other treatment conditions by exhibiting significantly higher “b*” values, indicating a unique effect on this specific colour quality (*p* < 0.05).

On the other hand, the powder that was stored at 40 °C in a transparent packaging displayed the lowest “b*” value among the conditions that were being studied, indicating a substantial difference between powder stored at 4 °C in a transparent packaging, storage at 25 °C with transparent packaging, and storage at 40 °C with transparent packaging. This distinct divergence indicates a perceptible departure in colour profile and reveals a noteworthy decrease along the yellow–blue axis within its colour features, in contrast to treatments with higher “b*” values (*p* < 0.05).

These findings are consistent with related research that, although conducted in different settings, examines how various treatments affect colour qualities. As an example, studies conducted by Hasizah et al. [24] reported that the dried moringa samples had positive b* values, which were considerably higher than those of the fresh sample. This suggests that the samples’ colour changed towards the yellow zone.

#### 2.1.4. Colour (h*)

Figure 2 indicates distinct trends in “h*” values among the various treatments, signifying notable differences in colour attributes. Specifically, the treatment stored at 40 °C with opaque packaging exhibited the lowest “h*” value among the assessed conditions, followed sequentially by storage at 25 °C with transparent packaging, storage at 4 °C with transparent packaging, storage at 25 °C with opaque packaging, storage at 4 °C with opaque packaging, and the initial baseline, denoted as the control (zero value). In contrast, treatment 40 °C in a transparent packaging showcased a higher “h*” value compared to 40 °C in opaque packaging, yet it registers lower values relative to storage at 25 °C with opaque packaging, storage at 4 °C with transparent packaging, storage at 25 °C with transparent packaging, storage at 4 °C with opaque packaging, and the control (zero value).

This observed hierarchy in “h*” values across treatments underlines the diverse impact of specific storage temperatures and packaging types on colour characteristics. The significantly lower “h*” value for the treatment stored at 40 °C with opaque packaging suggests a pronounced shift in colour attributes, potentially associated with the elevated storage temperature of 40 °C combined with dark packaging. Conversely, the treatment stored at 40 °C with transparent packaging demonstrates a relatively higher “h*” value compared to storage at 40 °C with opaque packaging, indicating a less intense alteration in colour characteristics, yet still positioned lower compared to several other treatments.

These findings align with comparable studies exploring the influence of storage conditions on colour attributes. Previous research by Hasizah et al. [24] has highlighted the subtle differences in colour characteristics linked to particular storage environments, which is consistent with the patterns shown in this study. The current work contributes to this understanding by elucidating the different orders of “h*” values among treatments, hence highlighting the different degrees of colour alteration produced by different combinations of temperature and packaging. This distinction draws attention to the intricate relationship that exists between colour characteristics and storage conditions, providing valuable insight into the optimization of packaging for the intended colour outcomes.

#### 2.1.5. Colour Difference “(ΔE)”

Figure 2 highlights the distinct variations in colour differences (“ΔE”) among the different treatments, indicating pronounced disparities in colour attributes. The results depict that samples stored at lower temperatures and in the dark resulted in minimal colour changes. On the other hand, the treatment stored at 40 °C with transparent packaging exhibited the highest colour difference among all assessed conditions, followed sequentially by storage at 40 °C with opaque packaging, storage at 25 °C with transparent packaging, storage at 4 °C with transparent packaging, and storage at 25 °C with opaque packaging. In contrast, the control (zero value) treatment and treatment stored at 4 °C in opaque packaging indicated no discernible changes in colour.

This observed hierarchy in colour differences underscores the diverse impact of specific storage temperatures and packaging types on colour attributes. The significantly elevated “ΔE” value at 40 °C with transparent packaging implied a substantial alteration in colour characteristics. Conversely, the absence of colour changes in zero value and 4 °C in opaque packaging indicated a consistent colour profile maintained under the respective storage conditions.

Similar trends have been identified in prior studies examining the influence of storage conditions on colour attributes. Research conducted by Ahad et al. [25] and Phahom et al. [20] have elucidated similar patterns, emphasizing the multifaceted impact of temperature and packaging variations on colour stability within diverse product contexts. Storage-induced alterations in dried leaves facilitate the colour transformation of chlorophyll, a phenomenon accelerated by heat and oxygen exposure [22]. Chlorophyll in leaves can experience oxidation, hydrolysis, and isomerization after harvest, which causes a change in colour from vivid green to olive green when two hydrogen atoms replace the magnesium atom in chlorophyll [25]. It has also been observed that increased polyphenol oxidase (PPO) activity speeds up chlorophyll deterioration in storage. It has also been observed that increased polyphenol oxidase (PPO) activity speeds up chlorophyll deterioration in storage [26].

By outlining the distinct sequence of colour changes among treatments and showing the various degrees of colour alteration caused by various combinations of packing and temperature, this study advances the understanding of colour changes in *M. oleifera*. This sophisticated understanding highlights the complex interactions that occur between colour characteristics and storage circumstances, highlighting the need for specific packaging techniques to regulate and preserve desired colour profiles. To fully understand colour stability and preservation in product packaging, more research into the mechanisms driving these colour variations is essential.

**Figure 3:**
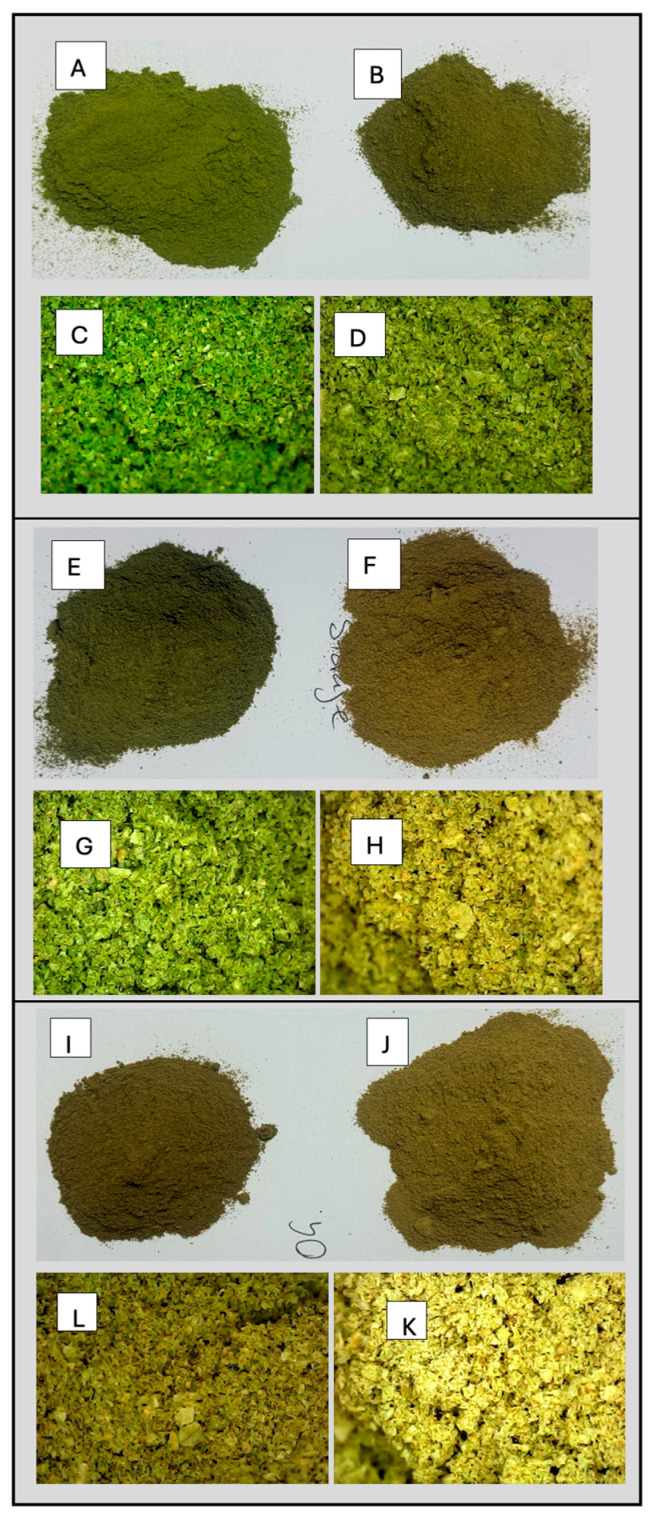
Effect of packaging and temperature treatment on colour of *Moringa oleifera* leaf powder as viewed and photographed using a Digital Canon EOS D70 15–135 mm lens Camera (EOSD70) and QY-X03 HD colour High-Resolution Digital Microscope (HDHRM). (**A**–**D**) Material storage at 4 °C [(**A**)—opaque packaging (EOSD70); (**B**)—transparent packaging (EOSD70); (**C**)—opaque packaging (HDHRM); (**D**)—transparent packaging (HDHRM)]. (**E**–**H**) Material storage at 25 °C [(**E**)—opaque packaging (EOSD70); (**F**)—transparent packaging (EOSD70); (**G**)—opaque packaging (HDHRM); (**H**)—transparent packaging (HDHRM)]. (**I**–**K**) Material storage at 40 °C [(**I**)—opaque packaging (EOSD70); (**J**)—transparent packaging (EOSD70); (**K**)—opaque packaging (HDHRM); (**L**)—transparent packaging (HDHRM)].

The photographs and microscope images presented in Figure 3 offer critical insights into the colour changes of the powder obtained through different storage methods. As depicted in Figure 3, opaque packaging exhibited better colour retention, with minimal fading, while higher temperature treatments in transparent packaging resulted in colour degradation. Most plant leaves, including *Moringa* leaves are rich in β-carotene. The compound is a key contributor to leaf pigmentation and easily degrades at elevated temperatures and light exposure. Hence, the transparent packaging combined with exposure to heat had a greater impact on the yellowness of *Moringa* leaves than the opaque packaging and lower temperature did.

### 2.2. Amino Acids

Table 1 provides data on the levels of various amino acids (His, Arg, Ser, Gly, Asp, Glu, Thr, Ala) in *M. oleifera* leaf samples subjected to different treatments. Amino acid concentrations fluctuated greatly depending on which treatment was given to the *M. oleifera* leaves. For instance, in comparison to the zero value (the control), the treatment stored at 25 °C with opaque packaging, 25 °C with transparent packaging, 40 °C with opaque packaging, 40 °C with transparent packaging, 4 °C with opaque packaging, and 4 °C with transparent packaging showcased alterations in the concentrations of these amino acids. Notably, certain treatments exhibited distinct trends in specific amino acids compared to others. Treatment storage at 25 °C with opaque packaging, for example, demonstrated lower concentrations of several amino acids (His, Arg, Ser, Gly, Asp, Glu, Thr, Ala) in comparison to the control. Conversely, the treatment stored at 40 °C with transparent packaging and the treatment stored at 4 °C with transparent packaging showed elevated concentrations of certain amino acids, indicating potential changes induced by the storage conditions and types of packaging used.

In addition, Table 1 highlights the concentrations of various amino acids (Pro, Lys, Tyr, Met, Val, Ile, Leu, Phe) in *M. oleifera* leaf samples subjected to different treatments, along with their respective mean concentrations ± standard deviation. Compared to the zero value (control), the treatment stored at 25 °C with opaque packaging, storage at 25 °C with transparent packaging, storage at 4 °C with opaque packaging, storage at 40 °C with transparent packaging, storage at 4 °C with opaque packaging, and storage at 4 °C with transparent packaging exhibit alterations in the concentrations of the specified amino acids. Each treatment displays distinct patterns in the concentrations of these amino acids relative to others. For instance, the treatment stored at 25 °C with opaque packaging demonstrated notably lower concentrations of several amino acids (Pro, Lys, Tyr, Met, Val, Ile, Leu, Phe) compared to the control sample. Conversely, the treatment stored at 40 °C with transparent packaging and the treatment stored at 4 °C with transparent packaging displayed increased concentrations of certain amino acids, indicating potential changes influenced by the storage conditions and packaging variations employed.

The changes in amino acid levels highlight how different packaging types (transparent and dark) and storage temperatures (25 °C and 40 °C) affect the amino acid content of *M. oleifera* leaves. These changes may have an impact on the functional traits and nutritional qualities linked to *M. oleifera* ingestion.

The distinct decrease in amino acid concentrations for the treatment stored at 25 °C with opaque packaging could be ascribed to several influential factors. Primarily, the storage conditions, encompassing a temperature of 25 °C in conjunction with dark packaging, likely fostered an environment that unfavourably impacted amino acid preservation. This is mainly through amino acid depolymerization and Maillard reactions [27]. This combination potentially led to degradation or breakdown processes, causing a reduction in amino acid concentrations over time. This observation aligns with prior research, indicating that extended exposure to elevated temperatures, particularly in the absence of light, may prompt degradation mechanisms that compromise amino acid stability [28,29].

Furthermore, the storage setting at 25 °C may have prompted oxidative reactions within the leaf samples. Oxidative stress, a consequence of environmental conditions, could instigate the degradation of amino acids, particularly susceptible ones like methionine (Met) and tyrosine (Tyr), thereby resulting in diminished amino acid concentrations [30]. This premise aligns with established findings that highlight the susceptibility of certain amino acids to oxidative damage under unfavourable storage conditions.

Moreover, the conducive nature of the dark storage environment, especially when coupled with moderate temperatures, might have facilitated microbial growth or enzymatic activity. This microbial or enzymatic metabolism could have contributed to the breakdown or utilization of amino acids, influencing the observed reduction in concentrations. Chemical interactions between amino acids and other compounds present in the storage milieu might have also transpired, instigating chemical reactions that altered or diminished certain amino acid levels. These interactions underscore the complex interplay of various compounds in storage environments, potentially impacting the stability of amino acids. Lastly, the duration of storage under these conditions likely played a pivotal role. Extended storage periods in suboptimal conditions could exacerbate degradation processes, culminating in the observed reduction in amino acid concentrations.

These theories are consistent with the body of research that has been conducted on the effects of storage conditions on the stability of amino acids in various matrices. Prior research has demonstrated associations between changes in the amino acid content of various biological samples and temperature, light exposure, oxidative stress, microbial activity, and chemical interactions [31].

### 2.3. Microbiological Analysis

Table 2 shows the microbial contamination assessments on powdered *M. oleifera* leaf samples subjected to the different storage conditions and packaging variations. The results indicated the presence and absence of various microorganisms, including *E. coli*, *Salmonella* sp., *S. aureus*, *B. cereus*, aerobic count, total coliforms, yeasts, and moulds across all treatments, including the control (zero value) and those subjected to varying temperatures (4 °C, 25 °C, 40 °C) and packaging types (opaque and transparent). There were also variations in the microbial contents depending on the storage conditions. Notably, *E. coli*, *B. cereus*, and *Salmonella* sp. were absent in all samples, suggesting the high level of hygenic practices during the preparation of the samples tested before and during storage. This observation indicated the safe use of the powder as these organisms were in the acceptable thresholds for food products.

Based on the results (Table 2), there were variations in the aerobic count (AC), total coliforms (TC), and yeast and mould (Y&M) for all the storage conditions with CFU/mL, slightly increasing as the temperature increase. From a food safety standpoint, the AC, TC, and Y&m counts for all the samples were below the limit set by South African National Standard (SANS1683:2015) Moringa Standard requirements.

Previous studies have indicated that *M. oleifera* possesses natural antimicrobial properties due to certain bioactive compounds [32]. However, despite these inherent properties, the microbial load observed in this study suggested that certain bacterial populations remained unaffected by the storage conditions and packaging variations studied. This implied the need for more rigorous processing or storage protocols to address these specific microbial populations, especially AC and TC, to ensure enhanced safety and quality of *M. oleifera* leaf products. For example, Olorade et al. [33] observed that glass material kept colour the best and was less prone to microbial permeability. Further investigations into the persistence of these bacteria despite storage conditions might aid in developing more effective preservation strategies for *M. oleifera* leaf products.

Although the results revealed that the storage conditions of *M. oleifera* leaf powder resulted in microbial counts below the SANS1683:2025 limit for *Moringa*, further isolation is recommended for colonies on the AC, TC, and Y&m for future studies on species-specific determination of these contaminants.

### 2.4. Phytochemicals

#### 2.4.1. Quercetin-3-Rutinoside

**Figure 4:**
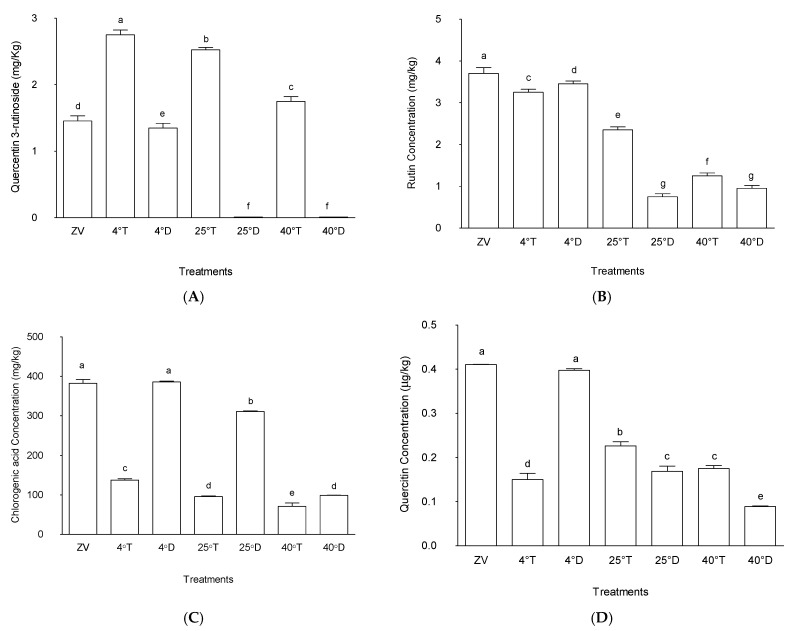
Effect of different storage conditions on (**A**) quercetin-3-rutinoside, (**B**) rutin, (**C**) chlorogenic acid, and (**D**) quercetin concentrations in *Moringa oleifera* leaves. ZV—zero-value baseline results of material analyzed before storage; 4 °T—storage at 4 °C with transparent packaging; 4 °D—storage at 4 °C opaque packaging; 25 °T—storage at 25 °C with transparent packaging; 25 °D—storage at 25 °C with opaque packaging; 40 °T—storage at 40 °C with transparent packaging; 40 °D—storage at 40 °C with opaque packaging. *n* = 6 per treatment. Results are expressed as the mean values ± standard error (*n* = 6) including control; bars in the same column marked with different letters indicate significance difference at *p* ≤ 0.05.

Figure 4 shows that treatment at 4 °C in a transparent packaging had the highest quercetin-3-rutinoside level in the grounded and stored *M. oleifera* leaves, with a mean value of 2.8 µg/g, showing statistical significance (*p* < 0.05) compared to the other treatments. This was followed by treatments at 25 °C in transparent packaging, which displayed a mean value of 2.46 µg/g, also significantly different from other treatments (*p* < 0.05). In contrast, treatments in opaque packaging at all the three storage temperatures (4 °C, 25 °C, 40 °C) exhibited lower levels of quercetin-3-rutinoside, with mean values of 0, 0, and 1.2 µg/g, respectively. The lower values of quercetin-3-rutinoside in opaque packaging suggest some form of impact of light during storage conditions on the preservation or degradation of this compound in *M. oleifera* leaves.

The zero value, representing the initial state of quercetin-3-rutinoside concentration in *M. oleifera* leaves before storage, showed an intermediate value of 1.23 µg/g. These findings underscore the possible biochemical activities that may continue despite the storage of plant material and, in this particular case, either accumulation or biosynthesis of quercetin-3-rutinoside in powdered *M. oleifera* leaves. Treatments subjected to transparent packaging allowing light tended to exhibit significantly higher concentrations, while those stored in opaque packaging with no light displayed lower levels. This suggests some compound biosynthesis are influenced by factors like light exposure or temperature fluctuations [34].

#### 2.4.2. Rutin

While quercetin-3-rutinoside concentration in powdered *M. oleifera* leaves during storage was affected by light conditions, the concentration of rutin varied according to the temperature. The treatment that exhibited the highest rutin concentration amongst the treatments was the control (zero value), representing the initial rutin concentration before storage, with a mean value of 3.76 µg/g (Figure 4). Following this, the treatment stored at 4 °C in both opaque packaging and transparent packaging exhibited rutin concentrations with mean values of 3.34 µg/g and 3.19 µg/g, respectively; both are significantly higher compared to other treatments (*p* < 0.05). These findings suggest that storage conditions at lower temperature, regardless of packaging type, notably preserved the rutin content in powdered *M. oleifera* leaves.

A decrease in the rutin concentration was observed in treatment at 25 °C with transparent packaging (mean value of 2.27 µg/g) compared to the treatment at 25 °C with opaque packaging (0.78 µg/g). Notable differences were also observed at 40 °C with opaque packaging versus the transparent packages, with the amounts being slightly higher in the latter than the former.

The results highlight the significant influence of storage conditions on rutin concentrations in *M. oleifera* leaves, with cooler storage conditions, specifically at 4 °C, demonstrating the highest preservation potential. These findings are consistent with other studies that have shown the impact of temperature on the stability and preservation of bioactive compounds in *M. oleifera* leaves. For instance, Kim et al. [35] observed that rutin content decreased at higher temperatures of up to 35 °C compared to storage at 4 °C. According to the authors, after two months at 4 °C, the rutin content did not significantly change. In contrast, the rutin contents were comparatively steady for two weeks at 25 °C and 35 °C before declining in the third week [35].

Rutin, a flavonoid glycoside found in plants, can degrade or reduce concentration when stored at high temperatures due to several reasons. First, rutin is heat-sensitive and can break down in high storage temperatures [36]. Second, elevated temperatures have the potential to induce oxidative stress in plant tissues. Rutin, an antioxidant molecule, may degrade in an effort to counteract free radicals produced in such stress situations [36]. Thirdly, the enzymes that break down rutin may become more active at higher temperatures [36]. The current findings underscore the importance of controlled storage conditions, particularly lower temperatures, in preserving bioactive compounds in *M. oleifera* leaves, such as rutin.

#### 2.4.3. Chlorogenic Acid

The effect of storage on chlorogenic acid concentration is presented in Figure 4. Storage at 4 °C with opaque packaging and the control (zero value), with mean values of 393.26 µg/g and 396.17 µg/g, respectively, indicated no significant difference between these two treatments (*p* > 0.05). Following this, the treatment stored at 25 °C with opaque packaging exhibited a relatively high chlorogenic acid concentration (mean value of 306.67 µg/g) compared to other treatments (*p* < 0.05). Conversely, the treatment stored at 40 °C with opaque packaging exhibited lower chlorogenic acid concentration, threefold lower than in the treatments in opaque storage at other temperatures. All the samples in transparent packaging had threefold lower chlorogenic acid concentrations. These findings suggest that storage conditions closer to ambient temperatures in opaque packages notably preserved chlorogenic acid content in *M. oleifera* powdered leaves.

These outcomes underscore the significant influence of storage conditions on chlorogenic acid concentrations in *M. oleifera* leaves. Previous studies have demonstrated that chlorogenic acid, found in *M. oleifera* has anti-inflammatory, anti-cancer, and antioxidant qualities [37]. Therefore, the preservation of chlorogenic acid content in *M. oleifera* leaves is important for maintaining its potential health benefits during storage.

#### 2.4.4. Quercitrin

A similar trend to the behaviour observed for chlorogenic acid was also observed for quercitrin concentrations. The observed quercetin concentrations in *M. oleifera* leaves under various storage conditions showed notable differences among treatments (Figure 4). The treatment stored at 4 °C in opaque packaging and the control (zero value) treatment exhibited the highest quercitrin concentrations, with a mean value of 400.4 µg/g and 397.3 µg/g, respectively. These were significantly higher than all other treatments. Conversely, treatments stored in transparent packaging at all temperatures as well as at 25 °C and 40 °C in opaque packaging showed lower quercitrin concentrations. These treatments demonstrated comparatively reduced levels of quercitrin compared to the treatment stored at 4 °C in opaque packaging.

The significant perseveration of quercitrin concentrations observed in the storage at 4 °C with opaque packaging treatments suggests that these storage conditions may be beneficial for maintaining its concentrations during the storage of *M. oleifera* leaves, hence preserving the plant leaf medicinal and antioxidant properties attributed to quercetin. Chaaban et al. [38] reported that quercitrin was prone to deterioration when exposed to light. Because quercitrin degrades easily when exposed to light, it is crucial to store plant-based products under ideal circumstances to prevent this degradation and maintain their quality.

These advancements in food preservation and processing align with the broader objective of Sustainable Development Goal (SDG) 2: Zero Hunger by 2030. By improving the nutritional quality and shelf life of food products, reducing post-harvest losses, and supporting sustainable agricultural practices, these techniques can contribute to improved food security and nutrition worldwide.

## 3. Materials and Methods

### 3.1. Plant Material

*Moringa oleifera* Lam. (Cultivar PKM1) fresh leaves were harvested and collected from the university orchard situated within the University of Limpopo in Limpopo Province, South Africa (coordinates: 23.8888° S, 29.7386° E). The first procedure entailed thoroughly cleaning and rinsing the fresh leaves with running deionized water. Following that, the leaves were defoliated, drained, and dried in an ambient room environment. The dried leaf materials were milled and sieved to generate a finely powdered form with particle sizes of 0.5 mm. The powdered substance was subjected to extensive testing to determine its colour attributes, phytochemical content, amino acid levels, and microbial contamination. This examination was performed initially and then again after 12 months of storage period to evaluate potential changes over time.

### 3.2. Experimental Design and Treatments

The experimental design used was a Randomized Complete Design (RCD) conducted in chambers with controlled temperature and environment to systematically assess the impact of various temperature storage conditions (4 °C, 25 °C, and 40 °C) and packaging types (opaque and transparent) on the colour attributes, phytochemical content, amino acid levels, and microbial contamination of the samples. The samples comprised 6 replicates per treatment at each storage temperature.

The trial layout was structured to accommodate three temperature storage conditions and two distinct packaging types, as follows: 4 °T—storage at 4 °C with transparent packaging; 4 °D—storage at 4 °C opaque packaging; 25 °T—storage at 25 °C with transparent packaging; 25 °D—storage at 25 °C with opaque packaging; 40 °T—storage at 40 °C with transparent packaging; 40 °D—storage at 40 °C with opaque packaging.

Additionally, the control sample, referred to as zero value (ZV) served as the baseline and was tested before the commencement of the storage period. Subsequent to the baseline assessment, the remaining samples underwent storage for a duration of 12 months under the designated temperature and packaging conditions. After the stipulated storage periods, the samples were subjected to testing to evaluate the alterations in their colour attributes, phenolic composition, and nutritional variation over time.

Photographs of the packaging material and the powder samples were taken using a Digital Canon EOS D70 Camera and QY-X03 HD colour High-Resolution Digital Microscope (Andowl, Jinhua, China).

### 3.3. Colour Measurement

The colour was measured using a Minolta Leaf CR-400 chromameter (Minolta, Osaka, Japan); the instrument had been calibrated by white tiles. The L* (lightness), colour coordinate (a*) related to the red and green colours where it had a positive or negative value, and (b*) values were determined, and the total colour difference (ΔE) was calculated using the formulae described by Managa et al. [39] previously. In the CIE colour system, negative a* colour coordinate and the positive b* colour coordinate describe the intensity of green and the yellow colour, respectively.

L1*, a1*, and b1* are day 0 values, while L2*, a2*, and b2* are the values of the sample treatments. Measurements were taken at three points per replicate. Altogether colour readings were recorded from two replicate samples per treatment.

Colour measurements were obtained for various *M. oleifera* packaging treatments, including temperature (4 °C, 25 °C, or 40 °C) and packaging type (Dark or transparent). The “Zero Value” served as the reference for colour comparison. Means and standard errors (SE) were calculated for each treatment group within the colour parameters (L*, a*, b*, h*). Delta E (ΔE) values were computed to quantify colour differences relative to the “Zero Value.” ΔE is a measure of how different the colour of each treatment is from the reference. Smaller ΔE values indicated closer colour matches, while larger values indicated greater colour differences.

### 3.4. Amino Acids Determination

The oven was set at 110 °C, and the protein/freeze dried tissue was added to a suitable glass sample vial. Then hydrochloric acid (6N HCl) was added to the vials containing the sample—between 0.3 and 1.0 mL. Samples were vortexed to make sure that all the sample material is submerged in the acid. The tubes were flushed with argon or nitrogen gas to eliminate oxygen, and then the tubes ware closed with the lids. Vials were placed in the oven, and after 5–10 min, the lids were tightened. The vials were left in the oven at 110 °C for 18–24 h. The vials were taken out of the oven then cooled. The hydrolysate was filtered using centrifuge tube filters (Corning^®^ Costar^®^ Spin-X tubes, Merk, Darmstadt, Germany). The filtrate was transferred to Eppendorf tubes and dried down using a speed vac, and then reconstituted in borate buffer to be ready for derivatization.

#### Derivatization Procedure

Sample dilutions were performed in 2 mL Eppendorf tubes, and 10 µL of diluted sample was then used during the derivatization. Borate buffer of 70 µL was transferred into a 200 µL glass and inserted in a 2ml glass vial. Then 10 µL diluted sample/standard solution was added thereafter. An amount of 20 µL of AQC reagent was also added. The vials were vortexed for proper mixing. Then, the vials were place into an oven/heating mantle at 55 °C and heated for 10 min. After 10 min, the vials were ready for analysis and were loaded onto the autosampler tray.

### 3.5. Determination of Microbial Contamination

The *M. oleifera* leaf powder samples were subjected to dilution series to obtain various dilutions in which 1g dissolved in 5 mL sterile distilled water. The dilution of each sample with aliquots were plated on agar plates. After 24-48 h, colonies were counted using the semi-automatic digital device. The microbial population was quantified in terms of bacterial count per millilitre (CFU/mL).

#### 3.5.1. Sample Dilution

The initial dilution was prepared by adding 1 mL of the sample to a 99 mL sterile saline solution, making a 1/100 (or 10^−2^) dilution. Bacteria were dispersed by mixing the dilution vigorously to dissolve any aggregates. An amount of 1 mL from the 10^−2^ dilution was moved to a second 99 mL saline solution, creating a 10^−4^ dilution. This procedure was continued until a 10^−8^ dilution was obtained.

#### 3.5.2. Plating and Incubation

One Petri dish received 1 mL of each dilution sample, while another received 0.1 mL. Nutrient agar was heated at a temperature of between 48 and 50 °C in a water bath which was then thoroughly blended to encourage bacterial growth for E. coli and total coliforms the samples. The presence of Salmonella in the samples was determined using the Xylose Lysine Deoxycholate (XLD) agar. Microbial growth was promoted by heating the plates and turning the oven to 25 °C for 48 h.

#### 3.5.3. Colony Counting

The treatment samples analyzed were 6 replicates per packaging at each storage temperature. Therefore, the total samples were 36 plus the 6 analyzed as baseline results to give 42 samples. The Petri plates with 30 to 300 colonies were taken for counting after incubation. Plates with more than 300 colonies were deemed too many for an accurate count, while plates with fewer than 30 colonies were deemed insufficient for a reliable statistical analysis. An automated colony counter was used to count the colonies on each plate (ZR-1101, Qingdao, China). The following equation was used to calculate the number of bacteria:

Number of bacteria (CFUs) = Dilution × Number of colonies on plate

### 3.6. Phenolic Variations

A 50 mL centrifuge tube with screwcap was used to accurately weigh the 2 g sample. An amount of 15mL of 50% methanol/1% formic acid was added and the tubes tightly capped. After that, the samples were centrifuged for 1 min, and then they were extracted in a vacuum bath for an additional hour. After 5 min, the 2 mL sample was taken and centrifuged at 14,000 revolutions per minute. The clear supernatant was then transferred into 1.5 mL glass vials for analysis.

A Waters Synapt G2 Quadrupole time-of-flight (QTOF) mass spectrometer (MS) connected to a Waters Acquity ultra-performance liquid chromatograph (UPLC) (Waters, Milford, MA, USA) was used for high-resolution UPLC-MS analysis. Before entering the mass spectrometer, column eluate went through a Photodiode Array PNPDA detector and was able to simultaneously obtain UV and MS spectral data. An electrospray ionization device was used in negative mode, with a cone voltage of 15 V, desolvation temperature of 275 °C desolvation gas at 650 Lph, and the rest of the MS settings optimized for best resolution and sensitivity. Data were acquired by scanning from *m*/*z* 150 to 1500 *m*/*z* in resolution mode as well as in MSE mode. In MSE mode, two channels of MS data were acquired, one at a low collision energy (4 V) and the second using a collision energy ramp (40−100 V) to obtain fragmentation data as well. For accurate mass determination, leucine enkaphalin was used as a lock reference mass and the instrument was calibrated in sodium format. Separation was achieved on a Waters HSS T3, 2.1 × 100 mm, 1.7 μm column. The injection volume was 2 L, and the mobile phase consisted of 0.1% formic solvent A and Acetonitrile which contained 0.1% formic acid as solvent B. For 1 min, the gradient started at 100% solvent A and gradually increased to 28% B over 22 min in a linear manner. The flow rate was set at 0.3 mL per minute, and the column temperature was maintained at 55 °C. The compound was quantified relative to the calibration curve set by injecting a range of catechin standards between 0.5 and 100 mg per litre for catechin.

Data was processed using MSDIAL and MSFINDER (RIKEN Canter for Sustainable Resource Science: Metabolome Informatics Research Team, Kanagawa, Japan). Generated data were subjected to statistical analysis using GenStat 18th version statistical package (VSN International, Hempstead, UK); to separate for significance among treatments, t-test was employed at the significance level of 5%.

## 4. Conclusions

Based on the results and objectives outlined in the current study, the following conclusions can be drawn. The storage temperature significantly influences the quality parameters of *M. oleifera* leaf powder. Variations in temperature conditions (4 °C, 25 °C, 40 °C) led to notable changes in phytochemical content, microbial activity, and nutritional properties over the 12-month storage period. Different storage material exhibited varying impacts on the phytochemical profiles, with higher temperatures and transparent packaging showing more pronounced degradation in bioactive compounds and increased microbial activity compared to lower temperatures. Lower temperatures (4 °C) in opaque packaging tend to better preserve the phytochemical constituents and nutritional attributes of *M. oleifera* leaf powder. This information serves as a valuable resource for stakeholders involved in the production, distribution, and consumption of this beneficial plant product. There was also notable increase in some small molecules from the possible heat- and light-assisted degradation of molecular compounds. Biological activities to determine the antioxidant, antimicrobial, and enzyme inhibition activities need to be performed to evaluate the effects of storage and observed chemical changes.

## Figures and Tables

**Table 1:** Effects of storage temperature and different packaging materials on amino acids of Moringa oleifera leaf powder.

	Treatments						
	ZV	4 °T	4 °D	25 °T	25 °D	40 °T	40 °D
Histidine	1.22 ± 0.001 ^a^	0.87 ± 0.020 ^cd^	0.94 ± 0.01 ^b^	0.89 ± 0.010 ^bc^	0.84 ± 0.020 ^cd^	0.8 ± 0.010 ^d^	0.86 ± 0.020 ^cd^
Arginine	2.44 ± 0.001 ^b^	2.38 ± 0.010 ^b^	2.07 ± 0.01 ^c^	2.52 ± 0.050 ^ab^	1.96 ± 0.040 ^c^	2.75 ± 0.050 ^a^	2.1 ± 0.050 ^c^
Serine	1.98 ± 0.020 ^a^	1.73 ± 0.050 ^b^	1.64 ± 0.01 ^b^	1.66 ± 0.020 ^b^	1.58 ± 0.020 ^b^	1.95 ± 0.040 ^a^	1.58 ± 0.010 ^b^
Glycine	2.65 ± 0.001 ^a^	1.86 ± 0.010 ^f^	1.96 ± 0.01 ^e^	2.03 ± 0.010 ^d^	1.93 ± 0.010 ^e^	2.18 ± 0.010 ^b^	2.11 ± 0.010 ^c^
Aspartic acid	4.78 ± 0.001 ^a^	3.15 ± 0.010 ^c^	2.56 ± 0.02 ^d^	2.45 ± 0.020 ^ef^	2.52 ± 0.010 ^de^	3.42 ± 0.020 ^b^	2.35 ± 0.020 ^f^
Glutamic	6.2 ± 0.001 ^a^	4.54 ± 0.020 ^c^	4.33 ± 0.02 ^d^	3.95 ± 0.010 ^g^	4.15 ± 0.010 ^e^	5.16 ± 0.010 ^b^	4.05 ± 0.020 ^f^
Threomine	2.1 ± 0.001 ^a^	1.6 ± 0.020 ^cd^	1.67 ± 0.01 c	1.56 ± 0.020 ^d^	1.63 ± 0.020 ^cd^	1.84 ± 0.001 ^b^	1.63 ± 0.020 ^cd^
Alanine	3.77 ± 0.001 ^a^	2.05 ± 0.010 ^c^	1.77 ± 0.01 ^e^	1.89 ± 0.010 ^d^	1.8 ± 0.010 ^e^	2.26 ± 0.010 ^b^	1.77 ± 0.010 ^e^
Proline	2.98 ± 0.001 ^a^	1.4 ± 0.010 ^c^	1.54 ± 0.05 ^bc^	1.54 ± 0.020 ^bc^	1.52 ± 0.010 ^bc^	1.6 ± 0.010 ^b^	1.59 ± 0.010 ^b^
Lysine	4.56 ± 0.001 ^a^	2.72 ± 0.020 ^f^	2.9 ± 0.01 ^e^	3.43 ± 0.020 ^c^	2.87 ± 0.010 ^e^	3.26 ± 0.010 ^d^	3.59 ± 0.010 ^b^
Tyrosine	2.44 ± 0.001 ^a^	1.22 ± 0.010 ^g^	0.97 ± 0.01 ^f^	0.54 ± 0.010 ^c^	1.04 ± 0.010 ^b^	1.12 ± 0.010 ^e^	0.63 ± 0.010 ^d^
Methionine	1.22 ± 0.001 ^a^	0.54 ± 0.001 ^d^	0.54 ± 0.01 ^d^	0.65 ± 0.001 ^c^	0.54 ± 0.001 ^d^	0.88 ± 0.001 ^b^	0.57 ± 0.001 ^d^
Valine	4.44 ± 0.001 ^a^	1.91 ± 0.010 ^de^	1.85 ± 0.02 ^e^	2 ± 0.020 ^c^	1.86 ± 0.020 ^e^	2.14 ± 0.010 ^b^	1.94 ± 0.010 ^cd^
Isoleucine	2.33 ± 0.001 ^a^	1.44 ± 0.010 ^c^	1.49 ± 0.01 ^c^	1.57 ± 0.010 ^b^	1.47 ± 0.010 ^c^	1.6 ± 0.010 ^b^	1.61 ± 0.010 ^b^
Leucine	4.33 ± 0.001 ^a^	2.6 ± 0.010 ^f^	2.83 ± 0.01 ^e^	2.89 ± 0.010 ^d^	2.89 ± 0.010 ^d^	3 ± 0.001 ^c^	3.13 ± 0.010 ^b^
Phenylalanine	5.33 ± 0.001 ^a^	2.92 ± 0.010 ^g^	3.2 ± 0.01 ^f^	3.75 ± 0.001 ^c^	3.28 ± 0.010 ^b^	3.52 ± 0.010 ^e^	3.96 ± 0.010 ^d^

ZV—zero-value baseline results of material analyzed before storage; 4 °T—storage at 4 °C with transparent packaging; 4 °D—storage at 4 °C opaque packaging; 25 °T—storage at 25 °C with transparent packaging; 25 °D—storage at 25 °C with opaque packaging; 40 °T—storage at 40 °C with transparent packaging; 40 °D—storage at 40 °C with opaque packaging. *n* = 6 per treatment. Results are expressed as the mean values ± standard error (*n* = 6) including control; same raw marked with different letters indicate significance difference at *p* ≤ 0.05.

**Table 2:** Effect of storage temperature and different packaging materials on microbial contamination of Moringa oleifera leaf powder.

Microorganisms (CFU/g)							
Treatments	E.c	S.Sp.	S.a	B.c	Ac	Tc	Y&m
ZV	Absent	Absent	Absent	Absent	<10	20	0.5 × 10^2^
4 °T	Absent	Absent	Absent	Absent	<10	33	<10
4 °D	Absent	Absent	Absent	Absent	10	33	<10
25 °T	Absent	Absent	Absent	Absent	20	50	<10
25 °D	Absent	Absent	Absent	Absent	40	55	<10
40 °T	Absent	Absent	Absent	Absent	60	56	<10
40 °D	Absent	Absent	Absent	Absent	40	50	<10
SANS1683:2015 Requirements	0	NS	NS	0	NS	≤1.0 × 10^2^	≤5.0 × 10^2^

ZV—zero-value baseline results of material analyzed before storage; 4 °T—storage at 4 °C with transparent packaging; 4 °D—storage at 4 °C opaque packaging; 25 °T—storage at 25 °C with transparent packaging; 25 °D—storage at 25 °C with opaque packaging; 40 °T—storage at 40 °C with transparent packaging; 40 °D—storage at 40 °C with opaque packaging. *n* = 6 per treatment. Results are expressed as the mean values ± standard error (*n* = 6) including control; same column marked with different letters indicate significance difference at *p* ≤ 0.05. E.c—*Escherichia coli*; S.a—*Staphylococcus aureus*; B.c—*Bacillus cereus;* S.Sp—*Salmonella Sp.;* Ac—aerobic count; Tc—total coliforms; Y&m—yeasts and moulds; NS = not stated.

## Data Availability

The research data is available upon request.
